# Hospital-based proton therapy implementation during the COVID pandemic: early clinical and research experience in a European academic institution

**DOI:** 10.1007/s12094-023-03127-3

**Published:** 2023-03-24

**Authors:** Felipe A. Calvo, Jacobo Palma, Javier Serrano, Mauricio Cambeiro, Rosa Meiriño, Santiago Martin, Diego Azcona, Diego Pedrero, Borja Aguilar, Jose Miguel Delgado, Verónica Moran, Alberto Viñals, Pablo Cabello, Elena Panizo, Alvaro Lassaletta, Carlota Gibert, Lidia Sancho, Jose Maria Fernandez de Miguel, Beatriz Alvarez de Sierra, Andres Alcázar, Victor Suarez, Alberto Alonso, Guillermo Gallardo, Javier Aristu

**Affiliations:** 1 Department of Radiation Oncology, Cancer Center, Clinica Universidad de Navarra, Madrid, Spain; 2 Department of Medical Physics and Radioprotection, Cancer Center, Clinica Universidad de Navarra, Madrid, Spain; 3 Department of Pediatric Oncology, Cancer Center, Clinica Universidad de Navarra, Madrid, Spain; 4 Department of Nuclear Medicine, Cancer Center, Clinica Universidad de Navarra, Madrid, Spain; 5 Department of Anesthesia, Cancer Center, Clinica Universidad de Navarra, Madrid, Spain; 6 Department of Radiology, Cancer Center, Clinica Universidad de Navarra, Madrid, Spain

**Keywords:** Oncology, Radiation oncology, Pediatric tumors, Medical physics, COVID-19

## Abstract

**Introduction:**

A rapid deploy of unexpected early impact of the COVID pandemic in Spain was described in 2020. Oncology practice was revised to facilitate decision-making regarding multimodal therapy for prevalent cancer types amenable to multidisciplinary treatment in which the radiotherapy component searched more efficient options in the setting of the COVID-19 pandemic, minimizing the risks to patients whilst aiming to guarantee cancer outcomes.

**Methods:**

A novel Proton Beam Therapy (PBT), Unit activity was analyzed in the period of March 2020 to March 2021. Institutional urgent, strict and mandatory clinical care standards for early diagnosis and treatment of COVID-19 infection were stablished in the hospital following national health-authorities’ recommendations. The temporary trends of patients care and research projects proposals were registered.

**Results:**

3 out of 14 members of the professional staff involved in the PBR intra-hospital process had a positive test for COVID infection. Also, 4 out of 100 patients had positive tests before initiating PBT, and 7 out of 100 developed positive tests along the weekly mandatory special checkup performed during PBT to all patients. An update of clinical performance at the PBT Unit at CUN Madrid in the initial 500 patients treated with PBT in the period from March 2020 to November 2022 registers a distribution of 131 (26%) pediatric patients, 63 (12%) head and neck cancer and central nervous system neoplasms and 123 (24%) re-irradiation indications. In November 2022, the activity reached a plateau in terms of patients under treatment and the impact of COVID pandemic became sporadic and controlled by minor medical actions. At present, the clinical data are consistent with an academic practice prospectively (NCT05151952). Research projects and scientific production was adapted to the pandemic evolution and its influence upon professional time availability. Seven research projects based in public funding were activated in this period and preliminary data on molecular imaging guided proton therapy in brain tumors and post-irradiation patterns of blood biomarkers are reported.

**Conclusions:**

Hospital-based PBT in European academic institutions was impacted by COVID-19 pandemic, although clinical and research activities were developed and sustained. In the post-pandemic era, the benefits of online learning will shape the future of proton therapy education.

## Introduction

Proton beam therapy (PTB) is an efficient high-precision radiotherapy technique developed in the context of: (1) a substantial increase in the global incidence of cancer; (2) an improved understanding of the biological basics of cancer; and (3) the innovative clinical management to further improve the therapeutic index: promotion of cancer control with minimal clinically relevant toxicity [1, 2]. In particular, technological progress in radiotherapy builds upon the Veronesi`s principle of evolutive oncology: “From maximum tolerated to minimal efficient treatment” [3].

A renewed interest in the normal tissue tolerance and protection in Radiation Oncology has grown around the technology, being able to generate a dramatic improvement in the dosimetric distribution of radiotherapy: only if a dosimetric benefit is achievable, a clinical benefit can be expected. PBT brings to daily practice the opportunity to explore clinical benefit in terms of exquisite normal tissue protection to unnecessary radiation and its impact in health economics and personal disability [4]. The very long-term effects of low and intermediate doses of radiation are only partially known. In pediatric patients treated with radiotherapy these adverse effects are consistently known when the survivors reach adulthood [5]. However, in adult patients, the long-term 20 years or more effects of radiation have not been systematically analyzed except for the description of substantial increments in general comorbidities [6].

Radiotherapy is an essential component of cancer therapy, rarely used alone with a curative intent. In the era of precision medicine, oncology is evolving to interdisciplinarity and molecular individualization [7, 8]. Progress in medical technologies applies the different medical specialties involved in diagnosis and treatment of each individual patient. Multidisciplinary Tumor Boards (MTD) are an essential part of an efficient approach to cancer management [9]. Patients treated under the recommendations generated of an MTB have significant impact on 5-year survival [10]. Human cancer is unique to each patient, and within the same patient (metastasis, primary site or recurrence) has its own biological pattern of progression and host adaptation pathways [11].

Hospital-based PBT is a particular structural model to facilitate, promote and assure multidisciplinary care to cancer patients [12]. The extra requirements of PBT facilities in terms of a large physical space, strict radioprotection legislation and patients’ accessibility, are elements to be solved within the complex scenario of an existing institution. Furthermore, from this “macro” view of PBT implementation (integration in the institution structure) comes the next challenge to be incorporate into a PBT program: the need for a “micro” solution for quality multi-specialty care. In high modern precision radiotherapy modalities, the interpretation of imaging studies and the definition of anatomical areas involved or at risk for cancer extension candidates for PBT, as well as the identification of normal uninvolved anatomic organs and structures are key to properly exploit exquisite dosimetric distribution together with minimal unnecessary irradiation to normal adjacent tissues. This “micro” solution is implemented by a team-work approach of radiation oncologists and imaging specialists from Radiology and Nuclear Medicine contributing with significant inputs not only at the time of individual contouring of PBT targets, but also with the preparation of the patient and the best images acquisition techniques for radiation planning. Additionally, PBT is a component of treatment in most cancer patients that will require multimodal therapy strategies. MTD discussion of each individual patient is mandatory in contemporary personalized oncology in the “micro” level of the decision-making process in clinical practice. Multi-specialist supported contouring and strategic consensus for multimodal therapy process are examples for optimized care promoted under the structure of a hospital based PBT program. In the “nano” level of optimized solutions for cancer patients candidates to PBT, some of the items to be integrated and provided by a hospital-based PBT practice include: the availability of anesthesia support, pediatric care, ICU backup, specialized nursing control, multi-specialists baseline evaluations for functional status before PBT (hematology, ophthalmology, neurology, endocrinology, neuropsychology, etc.), and imaging re-scheduling for re-planning. At present, basics standards for molecular oncology clinical practice requires the availability of systematic pathology evaluation with special emphasis in molecular profiling, which includes the need for tissue access by invasive procedures (re-biopsies and liquid biopsy technology).

### COVID-19 pandemic and PBT initial experience: unexpected implications

A rapid deploy of unexpected early impact of the COVID pandemic in Spain was described in 2020 [13]. As of Oct 12, there were 861,112 confirmed cases and 32,929 deaths due to COVID-19 in Spain. More than 63,000 health-care workers have been infected. Spain was one of the most affected countries during the first wave of COVID-19 (March to June 2020) and was also hit hard again by a second wave of COVID-19 infections. While the reason behind this outcome is pending of a long-term analysis to be fully understood, Spain's COVID-19 crisis indicated weaknesses in some parts of the health system and revealed complexities in under stress management conditions. Along 2020 and 2021 a major disturbance of social life and health-care standards were affected by the alarm, and impacted the processes inside institutions, medical practice and research, development, and innovative projects, including PBT. In the context of a pandemic stress, the health care system in Spain proved to be understaffed, under-resourced, and under strain. With 5.9 nurses per 1000 people, Spain has one of the lowest ratios in the EU (where the average is 9.3 per 1000 people), and too often relies on temporary contracts. Paradoxically, health indicators (such as life expectancy and healthy life expectancy) suggest that Spain overperforms, in the previous decade, with indicators better than would be predicted according to the country's socio-demographic index [14].

Oncology practice was revised by experts to assist discussion about the risks and benefits to facilitate decision-making regarding multimodal therapy for prevalent cancer types amenable to multidisciplinary treatment. The radiotherapy component was re-assessed to search for treatment options that should be considered by health care professionals more efficient in the setting of the COVID-19 pandemic, minimizing the risks to patients whilst aiming to maintain cancer outcomes. The tendency was to recommend hypofractionated schedules for radiotherapy as it was reported for rectal and breast cancer [15, 16]. In international academic PBT centers the challenge was to balance delivery of cutting-edge cancer treatments with appropriate mitigation and management of COVID-19 risk for both staff and patients and to facilitate communication across referring doctors [17].

At the time of major pandemic peak goals for protecting patients and staff from SARS-CoV-2 infection while continuing to deliver high-quality PBT were defined, including: (1) a zero-visitor policy, with exceptions for adults with neurocognitive impairments, very old patients, and pediatric patients; (2) entry-point control with separate patient and employee screening checkpoints; (3) hand hygiene and provided surgical masks; (4) social distancing; (5) implementing work-from-home strategies for administrative staff, physicists, dosimetrists, nurses, and radiation therapists (RTTs); (6) telemedicine workflows for clinical visits, patient education, and obtaining consent; (7) self-quarantine in traveling patients and while under treatment; (8) requirement for new patients to have a negative test result for COVID-19 before proceeding with the first appointment [18]

### PBT practice in a European academic hospital: description

Recommendations adopted in routine daily practice across Europe have been studied to understand the different selection methods applied in the European PBT centers: most patients treated with PBT are included in prospective data registration programs and/or in clinical trials [19]. Task force groups and international projects such as the European Particle Therapy Network (EPTN) and INfraStructure in Proton International REsearch (INSPIRE) seek to provide and increase the collaboration between European centers, defining dedicated work packages for prospective data registration and clinical trials [20, 21].

The COVID-19 pandemic has had an unprecedented impact on the European PBT institutions. Only the National Health Service in United Kingdom, in particular the UK Ocular Oncology Services has evaluated the impact on the adult eye cancer care in the top 4 months of pandemic incidence, reporting an increased caseloads of enucleation and stereotactic radiosurgery (*p* > 0.05), in comparison to fewer PBT delivery (*p* < 0.05) [22].

**Fig. 1:**
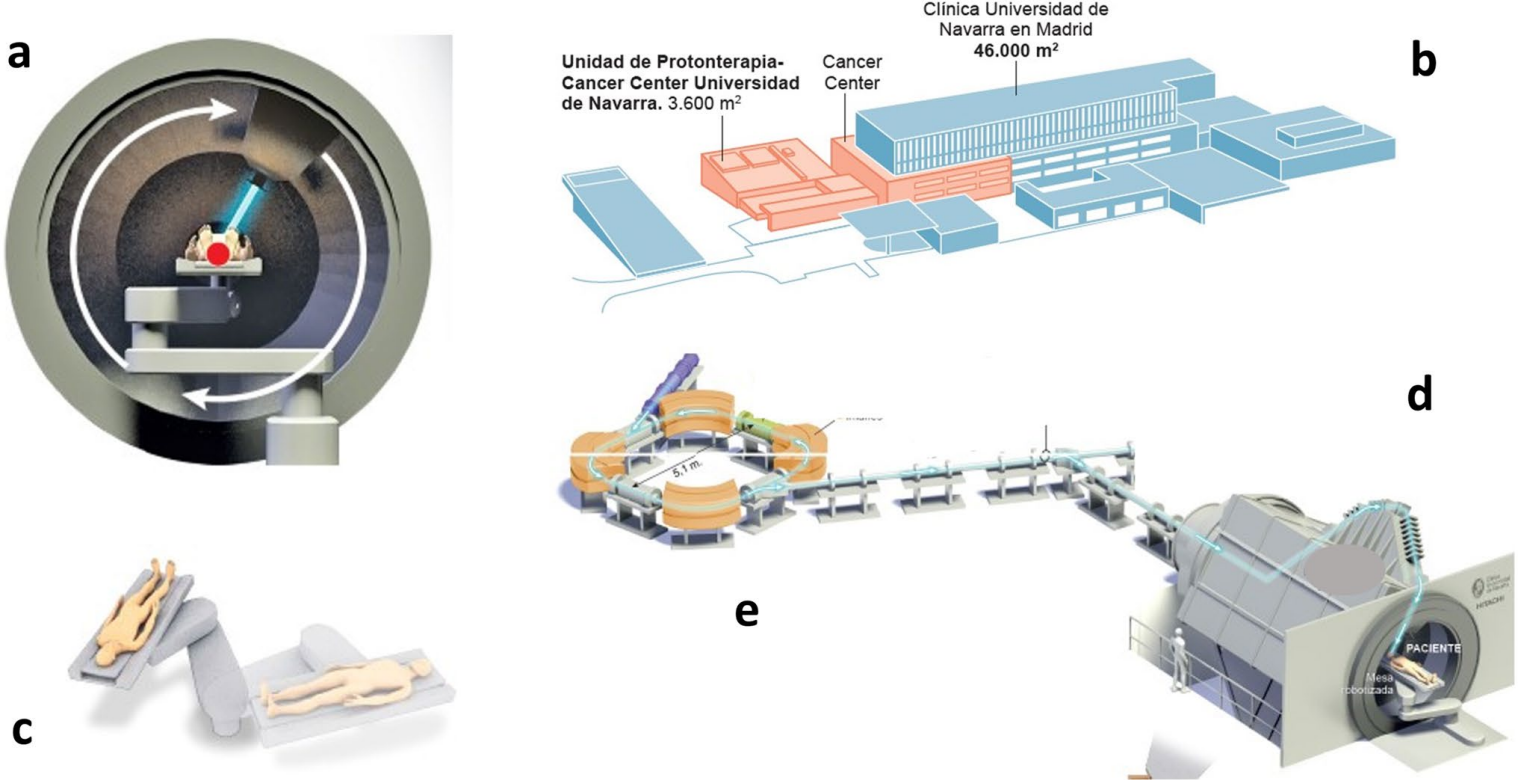
Illustration of structural and instrumental components of the configuration of the Proton Beam Therapy Unit at Clinica Universidad de Navarra (Madrid, Spain): **a** 360° gantry and cone-beam CT; **b** intra-hospital, Cancer Center area, PBT Unit implantation; **c** 6D treatment table; **d** beam line at the 360° gantry and treatment room; **e** synchrotron configuration

**Fig. 2:**
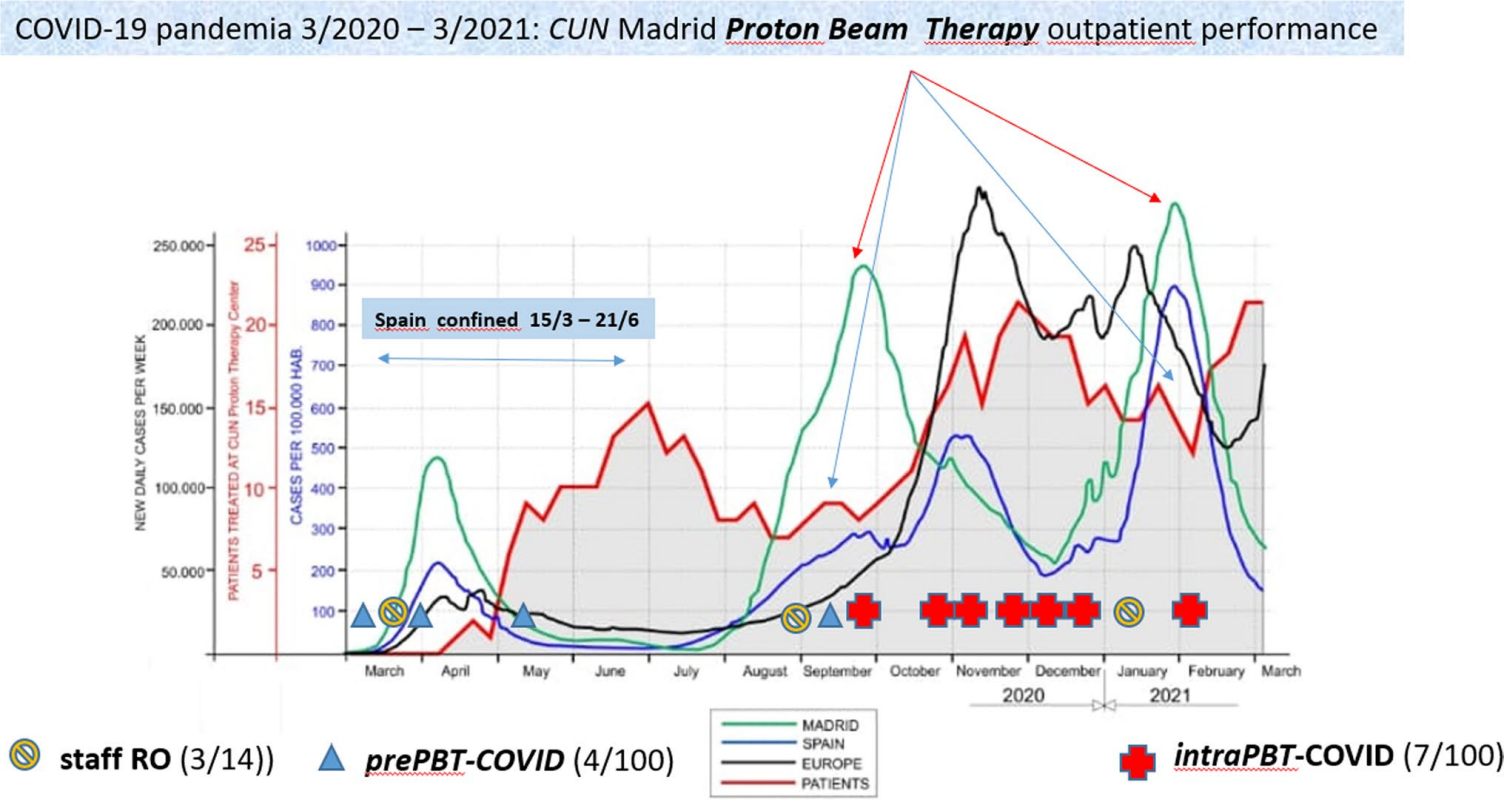
Chronology of COVID-19 pandemic incidence in Madrid, Spain and Europe, integrated with the rump-up of patients being treated at the Proton Beam Therapy Unit at Clinica Universidad de Navarra in Madrid (Spain) in the period march 2020 to march 2021. Incidence of COVID-19 positive test among staff-professionals and patients. *RO* radiation oncologist

In March 2020, after 28 months of construction, the first cancer patient was treated at the Clinica Universidad de Navarra Proton Therapy Unit in Madrid, Spain. This is the first commercial synchrotron equipment for PBT operating in Europe (Fig. 1) and the fourth commercial 360º gantry available for clinical use worldwide. It is important to emphasize that the initiation of clinical activities was coincident with COVID pandemic, in one of the cities in the world, Madrid, one of the most affected regions in terms of both cases and deaths (Spain). Under the strict institutional protective policy, for the period from march 2020 to march 2021, 3 out of 14 members of the professional staff involved in the PBT intra-hospital process have had a positive test for COVID infection. Also, 4 out of 100 patients had positive tests before initiating PBT, and 7 out 100 developed positive tests along the weekly mandatory special checkup performed during PBT to all patients (Fig. 2). It is noteworthy that the pandemic's peak incidence affected the number of patients undergoing PBT, and the difference in peak incidence's impact in Madrid and Spain, as most patients had to travel from peripheral regions for PBT.

An update of clinical performance at the PBT Unit at CUN Madrid describes the initial 500 patients treated with PBT in the period from March 2020 to November 2022. (Table 1). In November 2022 the activity reached a plateau in terms of patients under treatment and the impact of COVID pandemic became sporadic and controlled by minor medical actions. At present, the clinical data is consistent with an academic practice, based in the context of individualized consideration of optimized and best possible dosimetric solution for complex cancer patients, with the additional institutional and structural boosts of being a PBT Unit integrated in a University Hospital, together with pencil beam and 360º gantry technology. Relevant clinical and technical information is prospectively registered since the initiation of the activity (NCT05151952).

**Table 1:** Description of the initial 500 patients treated (period march 2020 to November 2022): patients, tumor and treatment characteristics

Initial 500 patients	
Patient characteristics	
N° patients	500 (100%)
Age (years)	
Median	48.8 (1–88)
< 30	168 (33%)
> 30	332 (66%)
> 75	45 (9%)
Gender	
Female	206 (41%)
Male	294 (58%)
Re-irradiation	
Yes	123 (24%)
No	377 (75%)
Concomitant QT	
Yes	143 (28%)
No	357 (71%)
Tumoral area	
Dermatological area	6 (1%)
Gynecological area	13 (2%)
Hematological tumor	4 (1%)
Hepato-bilio-pancreatic tumor	8 (1%)
Breast cancer	12 (2%)
Head and neck	64 (12%)
Pediatrics cancer	131 (26%)
Atypical teratoid rhabdoid tumor (ATRT)	5 (1%)
Craniopharyngioma	7 (1%)
Ependymoma	14 (3%)
Germinoma	9 (2%)
Medulloblastoma	31 (6%)
Rhabdomyosarcoma	14 (3%)
Ewing's sarcoma	12 (2%)
Others	39 (8%)
Prostate cancer	54 (11%)
Lung	13 (2%)
Sarcoma	102 (20%)
Chordoma/chondrosarcoma	72 (14%)
CNS	63 (12%)
High grade glioma	24 (4%)
Gastrointestinal tract	26 (5%)
Miscellaneous	4 (1%)
Proton beam technique	
IMPT MFO synchrotron	500 (100%)
Total doses (median, range)	58.7 (14.4–75.9)
< 30 Gy RBE	23 (4.6%)
30–50 Gy RBE	91 (18.2%)
> 50 Gy RBE	386 (77.2%)
Fractionation (median, range)	28 (3–40)
< 10	22 (4.4%)
10–20	128 (25.6%)
> 20	350 (70%)
Fractionation (median, range)	3 (1–11)
1	37 (7.4%)
2	202 (40.4%)
3	114 (22.8%)
≥ 4	147 (29.4%)

### PBT early scientific developments: research and innovation

Research and innovation define the identity of an academic European hospital with advanced technological resources. Since the initiation of activities at the PBT Unit the multi-professional team (oncologists, physicists, medical imaging specialists, pathologists and other supporting medical and translational specialists) have generated projects to integrate research and innovation activities in the workflow of the staff and allied professionals.

At the time of the start of the clinical activity, and in the most challenging moment of COVID-19 early impact in society, several research projects were funded by public and private institutions (Table 2). The topics to be investigated in the context of PBT included: proton radiobiology in cell culture models, radionecrosis, dosimetric estimations and effects on biomarkers detected in circulating blood, localized prostate cancer treatment, and international cooperation for quality and safety IT-driven models.

**Table 2:** PBT related research project funded for Clinica Universidad de Navarra investigadors selected in competitive private and public contest

Research topic	Funding institution	Code	Calendar
Prostate cancer	Instituto de Salud Carlos III (Spain)	PI20/01598	2020–2023
Radiobiology	Ministerio de Ciencia, Innovación y Universidades (Spain)	PID2019-104558RB-I00	2020–2024
Radionecrosis	Instituto de Salud Carlos III (Spain)	PI20/01531	2020–2023
Prostate cancer dosimetry	Asociación Española Contra el Cancer (Spain)	ERAPERMED2020-110-1	2020–2023
Hormesis post-radiation	Ministerio de Ciencia, Innovación y Universidades (Spain)	PID2019-110369RB-I00	2020–2023
Quality and safety IT control	Horizon 2020 European Union (EU)	H2020-SU-DS-2018-2019-2020	2020–2024
Vessels/blood as organs-at-risk	Siemens International (EU)	C00232756/PR20-00,445	2020–2024

### Clinical research based on 360° gantry synchrotron performance PBT technology

Several preliminary analyses of clinical experiences in PBT have been reported including:

- Prostate cancer patients: ten patients with organ confined PC underwent fiducial Carbone marker & SpaceOAR prior CT scan and mpMRI planning radiotherapy in RayStation Planning System CTV prostate and DILs were defined using T2-weighted, dynamic contrast-enhanced and diffusion-weighted. Prescription dose on CTV Prostate was 60 Gy in 20 (RBE factor 1.1) Eq. 2 Gy α/β 2 (75.0 Gy). Prescription dose (PD) in CTV DILs was 110% of the CTV (Prostate) prescription. Treatment was delivered with Pencil Beam Scanning technique. IGRT megavoltage cone beam was performed before each treatment. All patients received more than 95% of PD on CTV prostate and CTV DIL; median D95% CTV Prostate was 58 Gy and median D95% CTV DIL was 62.7 Gy. All patients met predefined rectal and bladder constraints. No acute G2 or higher rectal or urinary toxicity were registered [23].
- Practice in pediatric cancer patients: in the initial 23-month period 96 patients were registered. Median age was 8.4 years (range, 1.2–19.4) and 49% required anesthesia. The median number of days elapsed between the simulation and the start of the treatment were 13.5 days. The most frequent tumors were: 66% CNS and 23% sarcomas. Seven percent reirradiation status. Treatment modality consisted in focal radiation (69%), CSI (27%), WVI (3%), and WBRT (1%). Median dose was 54 Gy (range, 15–72 Gy) focal, and 36 Gy (range, 18–39.6 Gy) craniospinal. Thirty-six patients received concomitant chemotherapy. All patients completed their proton segment plan. The non-hematological toxicity grade ≥ 3 occurred in 6 patients and grade ≥ 3 hematologic toxicity was related to concomitant chemotherapy, CSI or both. During the pandemic, 8 COVID-19 infections were diagnosed, 6 asymptomatic and 2 with mild symptoms. The status at evaluation was: alive without disease 68.8%; active treatment 17.2%; alive with disease 9.7%; and deceased 4% [24].
- Practice in geriatric ages: from July/2020 to March/2022, 40 patients older than 75 years (median 78; range, 75–89) have been treated with PBT. The distribution by tumor location or organ was as follows: 13 (32.5%) prostate, 8 (20%) head and neck, 7 GI (17.5%), gynecological tumors 3 (7.5%), brain 3 (7.5%), lung 2 (5%) and others 4 (10%). The median dose administered was 60 Gy (range, 20–72 Gy). Three patients developed grade 3 or higher toxicity (all had previously received RT). With a median follow-up of 14.3 months, 38 (95%) patients are alive [25].
- Skull-base chordomas: up to December 2021, 23 patients with pathology confirmed skull-base chordoma were treated. Mean age was 45 years (range, 5–74 years). Twelve were men (52%). Most frequent histological type was conventional chordoma (70%). Most common location was central clivus in 13 cases (57%), followed by sphenoid (18%), and petroclival (13%). All patients underwent surgery. Macroscopic (R2) residual tumor persisted in 74% of the cases. Six patients (26%) received prior radical dose of photon irradiation (EQD2mean = 67.8 Gy), with a median time between radiotherapy treatments of 50 months (range, 15–126 months). Mean size of treatment CTV was 18.8 cc (range, 0.5–80.6 cc). Median prescribed dose was 72 Gy RBE in 30 fractions (EQD2 = 79.2 Gy RBE), with a mean V95 = 94.2%. The mean maximum doses D0.1 cc at OAR were: brainstem = 44.7, chiasm = 32.8, and optic-pathways = 31.3 Gy RBE. Dominant symptoms before IMPT were headache (44%), and diplopia (35%). No grade 3–4 toxic events were documented. With a 12 months median follow-up, 17 patients showed stable disease (74%), 4 radiological response (18%), 1 progression (4%), and 1 cardiovascular death (4%). Symptoms improved/disappeared in 21 patients (91%). The 1-year progression-free-survival was 95.5% [26].
- Dosimetric comparisons protons versus photons: eight patients diagnosed with lung (4) or esophageal (4) cancer treated with intensity modulated proton therapy (IMPT), were compared to alternative dosimetric plan with photons (VMAT). All the plans were evaluated with classical constraints. There was a statistical difference between IMPT & VMAT on V8Gy & V16Gy for heart & V5Gy & Dmean for lungs [27].
- Experience on selective re-irradiation. First 94 patients (median age 58 years) treated with proton-reirradiation (PBT-rRT) were analyzed. Most patients were referred from Spanish regions (80%). The distribution by tumor location or histology were: 28 (30%) CNS, 18 (19%) head and neck, 16 (17%) sarcoma, 9 (10%) GI tumors, 7 (7%) prostate, and 16 (17%) others. The median time elapsed between the first RT and PBT-rRT was 29 months (range, 3 m–470 m), and 60 Gy (range, 18 Gy–72 Gy) was the median initial RT-dose. The median CTV volume, PBT-rRT prescribed dose, number of fractions and dose fractionation were 70 cc (range, 2.5 cc–3769 cc), 54 Gy (range, 18 Gy–72 Gy), 20 fractions (range, 5–35), and 3 Gy (range, 1.5 Gy–7 Gy) respectively. The total EQ2 administered (initial RT + PBT-rRT) for a/b-3 and a/b-10 were 57.2 Gy and 54.7 Gy respectively. Twenty-six out of 52 patients experienced symptoms relief. Fifteen (16%) patients had acute toxicity grade 3 or higher. With a median follow-up of 13.4 months (range, 2 m–24.8 m), grade-3 or higher late toxicity was registered in 15 patients (16%). Six and 12 months OS was 91% and 84%, respectively [28].

### Translational research based on molecular imaging and blood circulating biomarkers

Specific data generated in the active research funded projects includes results on the potential of PET-CT 11-methionine/DOPA studies for improved contouring at the time of simulation and radiotherapy treatment planning to implement biology guided dosimetric prescriptions for pencil beam proton therapy technology [29]. Results in 17/17 patients diagnosed with primary or recurrent CNS tumors, showed areas of metabolism within the neoplasia range and showed a differential distribution of the tracer to guide boost delineation [30].

The research project more actively reported in our group is VASA (VAsos & SAngre; vessels & blood) [31, 32]. Craniospinal irradiation in medulloblastoma patients induces significantly less severe lymphopenia in children treated with protons compared to photons [33]. Furthermore, PBT increases levels of biomarkers related to vascular and endothelial radiation effects [34]. The search of vascular and immune biomarkers levels belongs to a work-in-progress long-term project assessing the development of a dose-volume-histogram applicable to standard clinical practice considering vessels and circulating blood as a very relevant organs-at-risk, in particular when alternative PBT is available. The potential of EDIC (the effective dose to circulating immune cells) as an independent predictive factor for hematologic toxicity is being investigated. Existing conventional imaging resources for dosimetric planning allow segmentation of vascular structures for accurate estimates of vascular DVH and blood dynamics. In our program, the most updated information generated in the period from November 2020 to April 2022, includes 54 patients treated with a radiotherapy component were prospectively analyzed in terms of dosimetric prescriptions. 4D MRI-flow sequences were exported for segmentation of the arterial and venous vascular tree up to a vessel diameter of 2 mm. Vascular and solid organ dose-volume histograms (DVH) were generated to calculate the EDIC as a ratio of blood flow based on the mean dose. In addition, the integral body and vessels dose were calculated by defining them as ID [Gy⋅L] = D [Gy]⋅V [L], where D [Gy] is the mean dose given to the volume V [L]. The mean PTV volume was 315 cc (± 157 cc). The mean vascular segmentation volume was 70 cc. The mean EDIC was 1 Gy. The mean body and vessel ID were 55 Gy*L and 1 Gy*L respectively. Frequency analysis showed that patients with higher EDIC correlated with vessel ID greater than 1 Gy*L. Quantifying irradiation exposure of circulating blood is feasible and requires the incorporation of high quality imaging of vascular structures for specific estimates of DVH and the implication in blood dynamics [32].

## Conclusions

Hospital-based PBT in European academic institutions was impacted by COVID-19 pandemic, although clinical and research activities were developed and sustained. In the post-pandemic era, the benefits of online learning will shape the future of proton therapy education and science transfer. An interesting platform is the online Christie Proton School as an effective framework for the future online resource to be complemented by hybrid approaches including face-to-face learning [35].

## Data Availability

Data availability is under the reponsability of Principal Investigator and institutional requirement.
